# Real-Time Geometric Parameter Measurement of High-Speed Railway Fastener Based on Point Cloud from Structured Light Sensors

**DOI:** 10.3390/s18113675

**Published:** 2018-10-29

**Authors:** Hao Cui, Qingwu Hu, Qingzhou Mao

**Affiliations:** 1 School of Remote Sensing & Information Engineering, Wuhan University, Wuhan 430079, China; cuihao@whu.edu.cn (H.C.); huqw@whu.edu.cn (Q.H.); 2 State Key Laboratory of Rail Transit Engineering Informatization (FSDI), Xi’an 710043, China; 3 Beijing Advanced Innovation Center for Imaging Technology, Key Laboratory of 3-Dimensional Information Acquisition and Application, Ministry of Education Capital Normal University, Beijing 100048, China

**Keywords:** high-speed railway, fastener geometric parameter measurement, structured light sensor, dense point cloud, region grow

## Abstract

With the increase in the number of service years for high-speed railways, the foundation of the rail track suffers from settlement, which causes rail track irregularity. To adjust the position of the track and meet track regularity demands, several components of the fastening system will be replaced by different sized components. It is important to measure the exact geometric parameters for the components of a fastening system before adjusting the track. Currently, the measurement process is conducted manually, which is laborious and error-prone. In this paper, a real-time geometric parameter measurement system for high-speed railway fastener based on 2-D laser profilers is presented. Dense and precise 3-D point clouds of high-speed railway fasteners are obtained from the system. A fastener extraction method is presented to extract fastener point cloud and a region-growing algorithm is used to locate key components of the fastener. Then, the geometric parameter of the fastener is worked out. An experiment was conducted on a high-speed railway near Wuhan, China to verify the accuracy and repeatability of the system. The maximum root-mean-square-error between the manual measurement and the system measurement is 0.3 mm, which demonstrates adequate accuracy. This system can replace manual measurements and greatly improve the efficiency of geometric parameter measurements for fasteners.

## 1. Introduction

**Figure 1:**
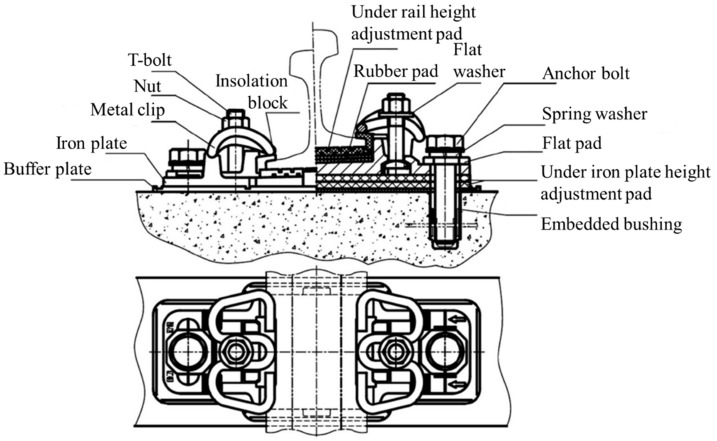
Assembly diagram of the WJ-7 fastening system [6].

Fasteners are critical parts of high-speed railway system, as they can fasten the rail track onto the track slab and maintain the track gauge. A typical high-speed railway fastening system (Figure 1) consists of a metal clip, bolts, an insulation block, a support pad and a height adjustment pad. The rail track of high-speed railway need to be adjusted accurately to ensure the high demands of track regularity [1,2]. During the adjustment of the track, the insulation block and the height adjustment pad may be replaced by those of different sizes according to the track adjustment plan [3,4,5]. Therefore, it is crucial to identify exact geometric parameters of the fastening system before adjusting the track. WJ-7, WJ-8, and Vossloh-300 are most commonly used type of fastener in China’s high-speed railways. Currently, the geometric parameter of the fastening system is measured manually, which is laborious and error-prone. Therefore, an effective and precise geometric parameter measurement system for high-speed railway fasteners is urgently needed. Since the minimum size difference between same fastener part of different specifications is 1 mm, the root-mean-square error (RMSE) of measurement should be less than 0.5 mm. 

In recent years, many researchers have developed fastener inspection systems based on images [7,8,9]. Yang et al. [10] presented an efficient method to detect fasteners based on image processing and pattern recognition techniques, which can be used to detect the absence of fasteners on corresponding tracks at high-speeds (up to 400 km/h). Khan et al. [11] proposed a machine vision-based technique to automatically detect the presence of rail line anchors/fasteners using Shi-Tomasi and Harris-Stephen feature detection algorithm. Gibert et al. [12] proposed an image-based method for fastener detection by using the histogram of oriented gradients features and a combination of linear SVM classifiers. Feng et al. [13] presented an automatic visual inspection system for the detection of partially worn and completely missing fasteners using a probabilistic topic model. Their method was able to simultaneously model diverse types of fasteners with different orientations and illumination conditions using unlabeled data. The systems mentioned above can detect fastener failure, but they cannot measure the geometric parameter of the fastener because images only provide two-dimensional information. To resolve this issue, three-dimensional information of the fastening system should be obtained. 

Structured light method is a promising way to achieve three-dimensional information acquisition. A structured light sensor consists of a structured light projector and a camera. The structured light projector generates a structured light pattern on the target, and the pattern is acquired by the camera. When the shape of the target changes, the shape of the projected-light pattern changes, as seen from the camera. Accurate profiles of the target can be computed by various structured light principles and algorithms [14]. The scanning range of the structured light sensor is shorter (less than several meters), and its precision is higher (submillimeter) than those of conventional three-dimensional information acquisition techniques, such as Light Detection and Ranging (LiDAR). The structured light method has been applied in many areas, such as 3-D visualization [15,16], railway track measurement [17,18,19,20,21], and component quality inspection [22]. Kjellander et al. [23] built a 3-D measurement system by mounting a structured light sensor on the arm of an industry robot. The 3-D point cloud of the target was acquired once the movement of the arm and a new planar segmentation algorithm was developed to extract planar surfaces from the target. Zhang et al. [24] proposed a structured light method based on motion images (SLMMI) to inspect missing fastener components. By using the SLMMI and the recognition method based on a neural network, the experiment achieved adequate results regarding speed and accuracy. Mao et al. [25] proposed a method to detect damaged and loose fasteners of high-speed railway based on structured light sensors. They proposed a centerline extraction method to extract the centerline of fastener’s metal clip and loose fasteners can by precisely detected by the extracted centerline. Lorenta et al. [26] built a system to detect rail gauges and missing rail fasteners based on reconstructed 3-D point cloud from structured light sensors. Rail gauges are estimated by RANSAC-based line detection and missing rail fasteners are detected by template matching method.

In this paper, a real-time geometric parameter measurement system for high-speed railway fasteners based on structured light sensors is presented. Dense and precise 3-D point cloud of high-speed railway fasteners are obtained from commercial structured light sensors (Keyence LJ-V7000 (https://www.keyence.com/products/measure/laser-2d/lj-v/index.jsp)). The posture and relative position of the structured light sensors are calibrated. The point cloud of the key area for the measurement of the fastener geometric parameter is extracted based on the original design of the fastening system and a region-growing method. Afterwards, the geometric parameter of the fastener is calculated according to its layout. The experiment was conducted on high-speed railways near Wuhan, China to test the performance of the system.

The rest of this paper is organized as follows: Section 2 provides an overview of the fastener measurement system. All of the methodological steps of the proposed approach are explained in Section 3. Section 4 explains the experiment and the results. Conclusions are drawn in Section 5.

## 2. Overview of the Measurement System

**Figure 2:**
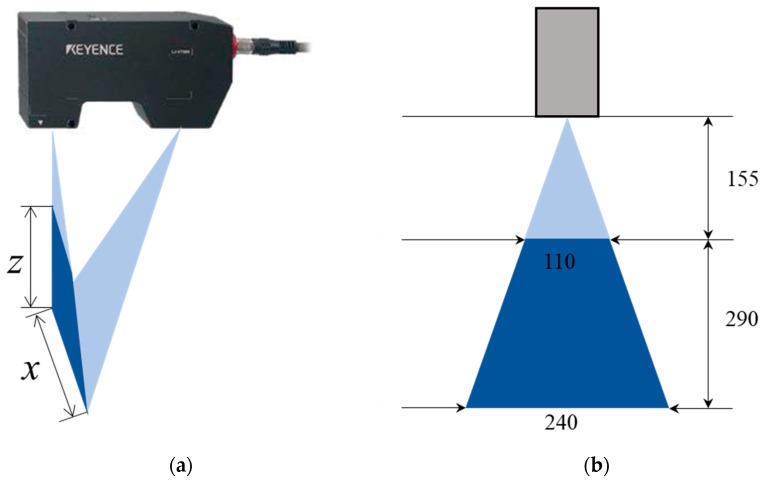
Structured light sensor (Keyence LJ-V7000). (**a**) Measurement coordinate; (**b**) Measurement range (millimeter).

Four commercial structured light sensors (Keyence LJ-V7000, Osaka, Japan), shown in Figure 2, are used in the measurement system. The sensors can provide a maximum of 800 points per profile and a maximum of 4000 profiles per second. Not only are the sensors able to record and output the distance information for each point (*Z*-axis), but they are also able to pinpoint the exact position of each point on the laser line (*X*-axis). The scanning range of the sensors is 110 mm at their minimum scanning distance 155 mm and can reach 240 mm at their maximum scanning distance 445 mm. The measurement range of the structured light sensors used in our system is shown as navy-blue isosceles trapezoid in Figure 2b. The repeatability of the sensors along the *Z*-axis and *X*-axis is 5 μm and 60 μm, respectively. The linearity is ±0.15%. The structured light sensor should be connected to an external controller when operating, and the controller can control two sensors simultaneously.

**Figure 3:**
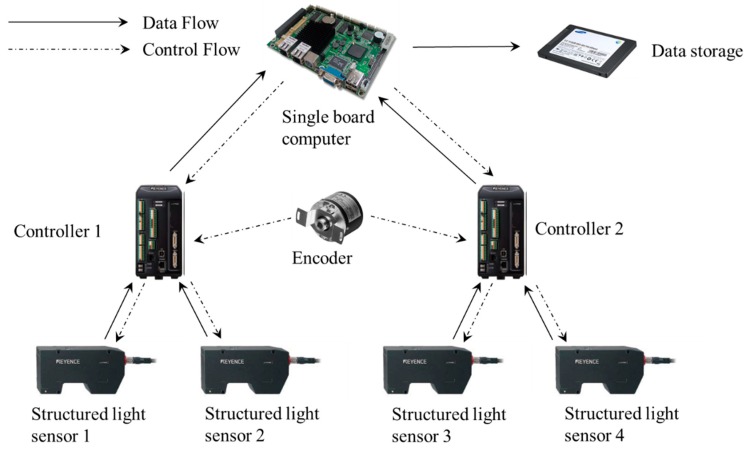
Architecture of the measurement system.

**Figure 4:**
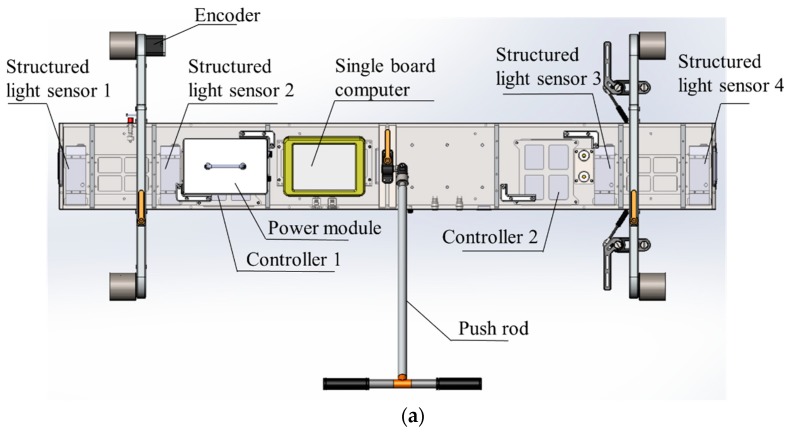

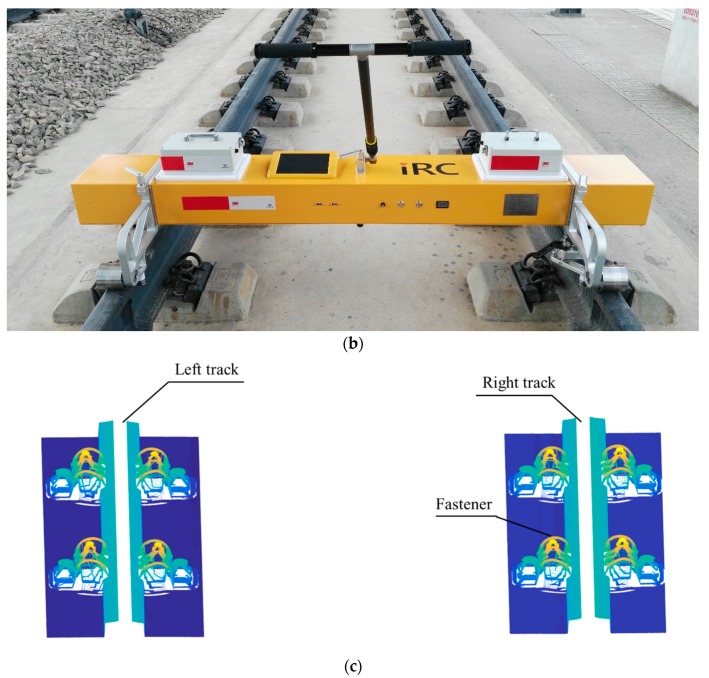
IRC system: (**a**) layout; (**b**) overview; (**c**) original output.

The measurement system is called the Intelligence Rail Checker (IRC), and its architecture is shown in Figure 3. The IRC consists of four structured light sensors, two controllers, a single-board computer (Intel ATOM D2550@1.86 GHz), a data storage module, an encoder, and an auxiliary power module. Communication and data transmission between the structured light sensors, controllers, single-board computer and data storage module are through Ethernet. The structured light sensors are triggered by an encoder. The online processing program is implemented into the single-board computer to enable real-time output. After online processing, the data from the structured light sensors are stored in the data storage module for further analysis. The layout of the IRC system is shown in Figure 4a. These modules are mounted on a detachable mechanical trolley, which allows them to be directly pushed forward onto the rail track. When the system is running on the rail track, the structured light sensors are triggered by the encoder every millimeter along the track. Then, the point cloud of the objects below the structured light sensors, including the track pad and fasteners, can be captured (Figure 4c). The point cloud is extremely dense (330 points/cm^2^) and precise, laying a solid foundation for the geometric parameter measurement of the fastening system.

## 3. Methodology

### 3.1. Calibration of the Structured Light Sensors

**Figure 5:**
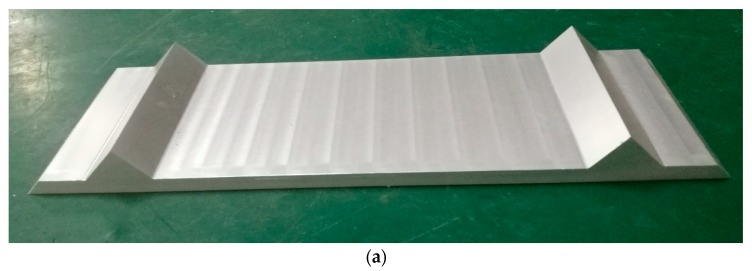

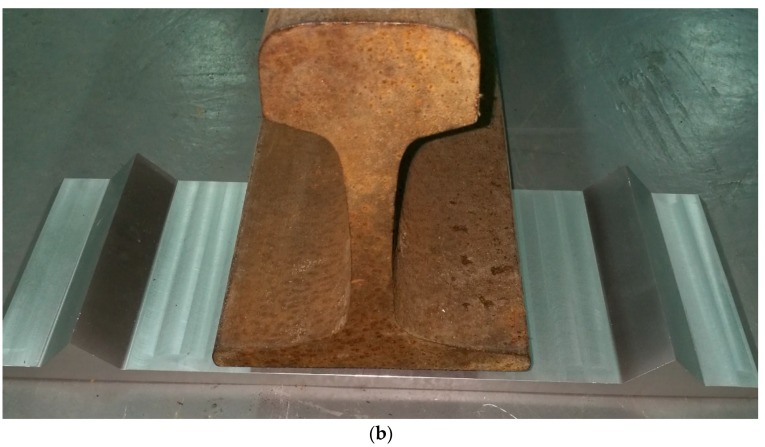
Special made calibration block for IRC: (**a**) full view of the calibration block; (**b**) how the calibration block is placed during calibration process.

In the original design of the IRC, the installation angles of the structured light sensors are perpendicular to the horizontal plane, and the four sensors are installed at the same height. However, due to installation errors, the actual positions and postures of the structured lighted sensors may be different from the original design. To calibrate the actual positions and postures of the sensors, a special made calibration block is designed, as shown in Figure 5.

The calibration block is processed from a single piece of aluminum alloy by a Computer Numerical Control (CNC) milling machine. Its two ridges are parallel and the upper horizonal surfaces are on the same plane. During the calibration process, the calibration block is placed horizontally, and its two ridges are parallel and symmetrical with the rail track. In addition, the rail tracks should be smooth and without defects, and the top surfaces of the two rail tracks are at the same height to ensure that the IRC is horizontal.

**Figure 6:**
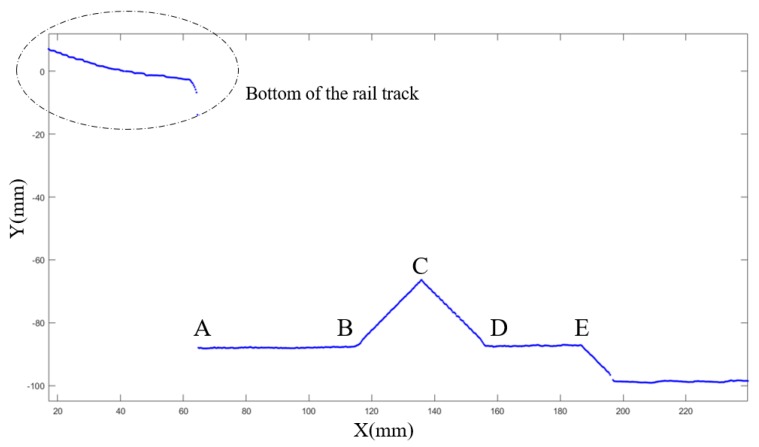
Point cloud of calibration block from structured light sensor.

When the IRC runs over the calibration blocks, the point cloud of the calibration blocks is generated by the structured light sensors. The original output frames of the structured light sensors contain segments of the calibration block, as shown in Figure 6. In this figure, points in the ellipse are bottom of the rail track and the rest points are ground and part of the calibration block. Segment *BC*, *CD*, and *ABDE* (segment *AB* and *ED* are on the same plane as mentioned above) are fitted to lines. According to the least-square fit, the fitting line for each segment can be worked out by Equations (1)–(3). Equation (1) is the equation of the fitting line, and the values of *a* and *b* can be obtained by Equations (2) and (3). In Equations (2) and (3), *n* represents the number of points in the segment, and $x_{i}$ and $y_{i}$ represent the *x* and *y* coordinate values at point $p_{i}$. Then, the intersecting segments (i.e., the ridges of the calibration block) can be worked out. According to the design diagram of the calibration block, the 3D posture and relative position between two consecutive structured light sensors can by calculated.

(1) y=ax+b

(2) $$a=\frac{\left(\sum x_{i}^{2})(\sum y_{i})-(\sum x_{i})(\sum x_{i}y_{i}\right)}{n(\sum x_{i}^{2})-{\left(\sum x_{i}\right)}^{2}}$$

(3) $$b=\frac{n(\sum x_{i}y_{i})-(\sum x_{i})(\sum y_{i})}{n(\sum x_{i}^{2})-{\left(\sum x_{i}\right)}^{2}}$$

**Figure 7:**
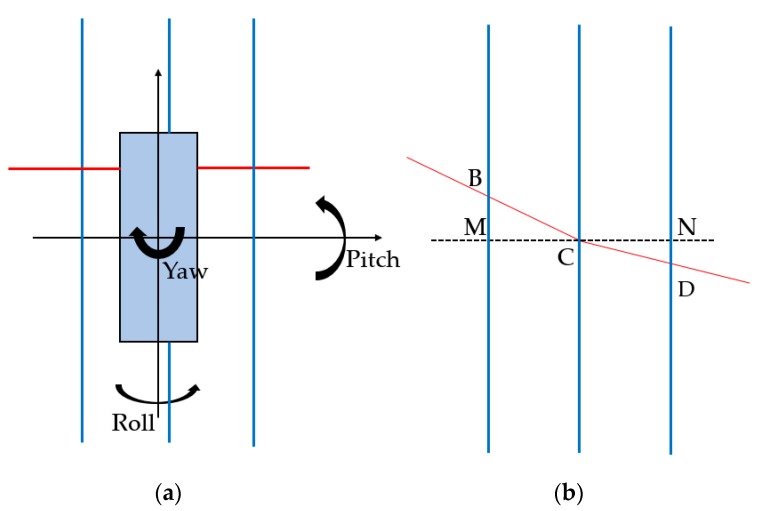
Illustration of 3D posture calibration of structured light sensors: (**a**) top view; (**b**) schematic diagram.

Figure 7 is the illustration of 3D posture calibration of structured light sensors. Figure 7a is the top view of a structured light sensor scanning the calibration block, the black angles indicate Euler angles of the sensor, the red line shows the scanning laser line of the sensor, and blue lines represent edges of wedge in the calibration block. Figure 7b is schematic diagram of scanning laser line and wedge. Segment *MCN* is an auxiliary line that perpendicular to the wedge. Segment *CB* and *CD* are the laser line on the surface of wedge, their deviation between *MCN* are caused by yaw and pitch of the sensor. ∠MCB is equal to yaw plus pitch while ∠DCN is equal to yaw minus pitch. Yaw and pitch of the sensor can be calculated through Equations (4) and (5), where α, β denotes yaw and pitch respectively. Roll of sensor’s Euler angles can be work out by calculating the slope of segment *ABED* in Figure 6.

(4) $$\alpha =(\arccos(\frac{MC}{BC})+\arccos(\frac{CN}{CD}))/2$$

(5) $$\beta =(\arccos(\frac{MC}{BC})-\arccos(\frac{CN}{CD}))/2$$

The detailed calibration steps are as follows:

(i) Choose two frames of the point cloud, where the frame number is the same for two consecutive structured light sensors, and put the two frames into the same two-dimensional Cartesian coordinates directly;
(ii) Select points in the same segment and calculate the fitting line;
(iii) Calculate the intersections among the fitting lines and work out the 3D postures of the two sensors according to the intersections;
(iv) Add 0 as the third dimension of frame points and rotate the point cloud, with (0,0,0) as the origin, and rotate frame points according to the 3D posture;
(v) Keep the point cloud for one sensor still and move the point cloud of the other sensor according to the design diagram of the calibration block so that the relative position between the two consecutive sensors can be obtained.

### 3.2. Fastener Point Cloud Extraction

The raw data from the structured light sensors contains point cloud for fasteners and other irrelevant components, such as rail pads, signal cables, and electrical devices. The point cloud for a fastener has to be extracted before the geometric diameter measurement process. To accomplish this, we propose a quick fastener point cloud extraction method.

**Figure 8:**
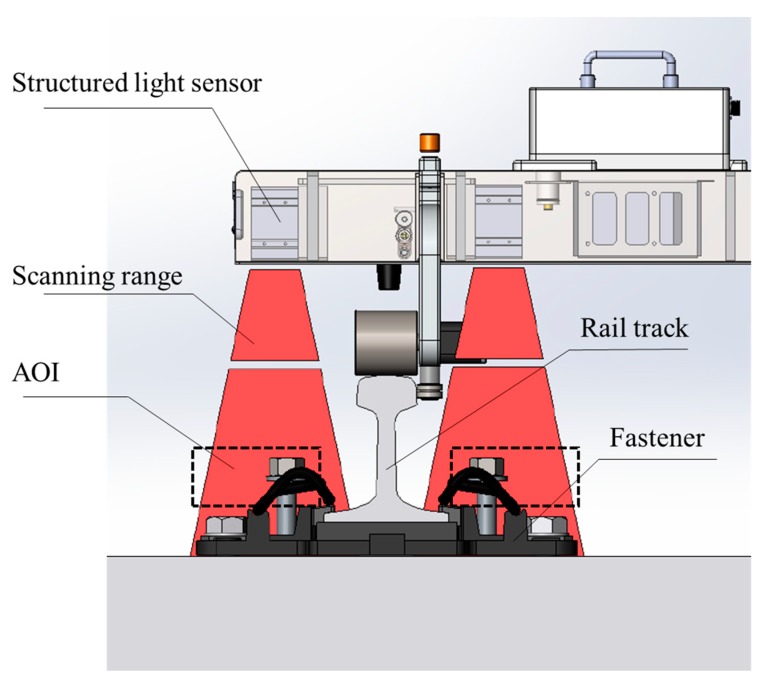
Illustration of the fastener extraction method.

There is an obvious feature in the raw data: Fasteners are higher than other parts of the rail tracks. Based on this feature, a method based on the point heights in a certain area is proposed. An illustration of the fastener extraction method is shown in Figure 8. The dotted box in this figure represents the area of interest (AOI), and the coordinates and size of the AOI are specifically selected to ensure that no other parts enter the area, except for the fastener. If any point in a frame enters the AOI, this frame is considered to a fastener-containing frame. After that, if several consecutive frames are all comprised of fastener-containing frames, then these frames comprised a fastener.

**Figure 9:**
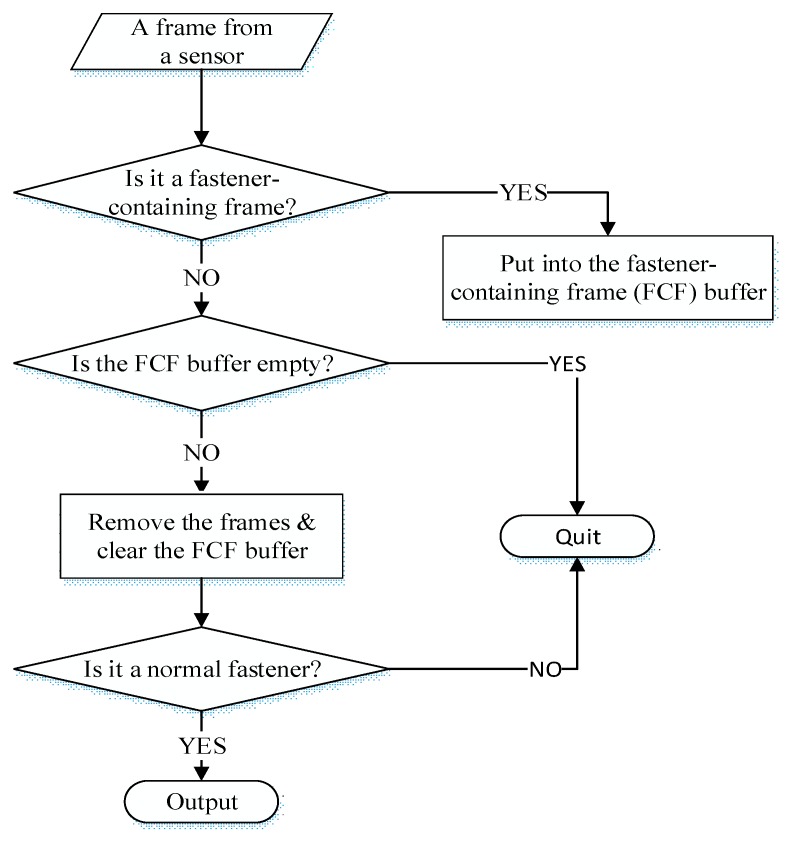
Overall steps of fastener extraction method.

Figure 9 shows the overall steps of the fastener extraction method. First, a frame from the structured light sensor is examined to determine whether or not it is a fastener-containing frame. If the frame is a fastener-containing frame, it is placed into the fastener-containing frame (FCF) buffer. If not, the FCF buffer is examined next. If the buffer is empty, this frame is deleted. If there are frames in the buffer, these frames are removed, and the buffer is cleared. The removed frames are considered as fasteners. Sometimes, irrelevant equipment next to the rail track, such as signal cables and electrical devices, falls into the AOI, which causes “fake fasteners”. To exclude these irrelevant parts, these frames will go thought normal fastener check. By checking the number of frames, average height, and height variance of the points, these fake fasteners can be excluded. After these processes, the point cloud of the normal fasteners is produced. These steps loop until all of the frames are checked. In detail, there are 800 points in a single frame, the distance between consecutive points in a single frame is 0.3 mm, and the distance between consecutive frames is 1 mm. The fastener extraction method is simple and efficient, it runs on the single board computer of IRC in real time. 

**Figure 10:**
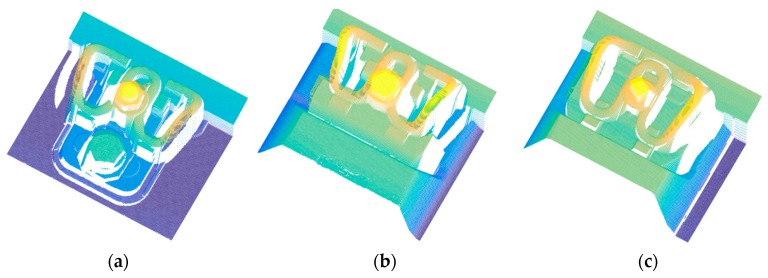
Extracted point cloud of the most commonly used fasteners in high-speed railways in China: (**a**) WJ-7; (**b**) WJ-8; and (**c**) Vossloh-300.

Figure 10 shows the extracted point cloud of the WJ-7, WJ-8, and Vossloh-300 fasteners, which are the most widely used fasteners in high-speed railways in China. The reflected light of the laser line pattern is blocked by the protruding parts of the fasteners, which cannot be received by the camera of the structured light sensor. As a consequence, some areas in the point cloud of the fasteners are blank.

### 3.3. Key Components Positioning

After the fasteners are extracted from the raw data from structured light sensors, their point clouds are sent to the single board computer for real-time analysis. Several fastener components, such as the insolation block and the height adjustment block, are obscured by other parts of the fastener, making it impossible to measure the geometric parameter directly. Therefore, indirect measurement based on the structure of the fastening system is ideal. To achieve this, several key components of the fastening system, such as the anchor bolt, iron pad and rail track, must be positioned. 

**Figure 11:**
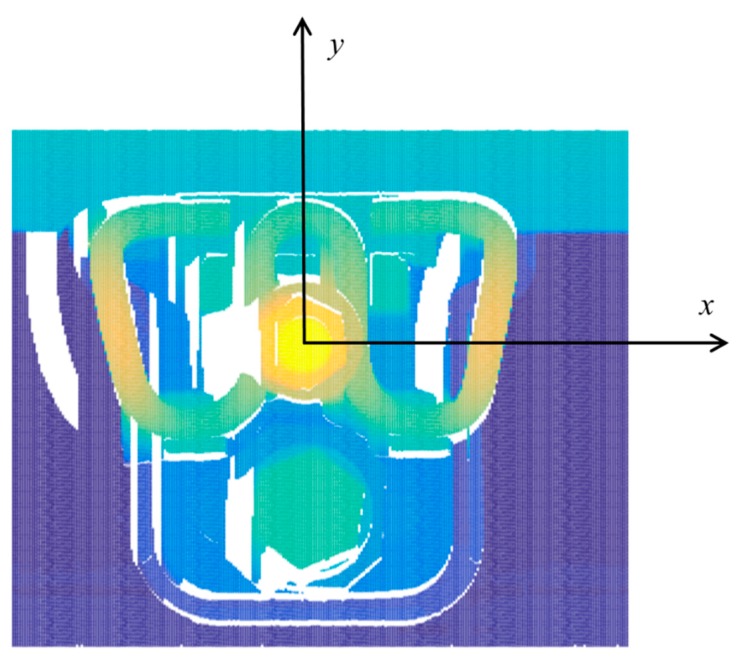
The coordinate system of a WJ-7 fastener.

The first step is defining the coordinate system of the fastener. The distance between the consecutive frame of the fastener’s point cloud is 1 mm, and there are a fixed number of points in a single frame. Therefore, the point cloud of a fastener can be placed into a matrix, where the column of the matrix indicates the frame number of a point, the row of the matrix represents the location of a point in the frame, and the value of the matrix represents the height value of the point. This is essentially similar to a digital elevation map (DEM). As mentioned above, WJ-7, WJ-8, and Vossloh-300 are the most used fasteners in high-speed railways in China. These three types of fastening systems have one characteristic in common: They all have a middle bolt, which is the highest part of the fastening system. Therefore, it is easy to locate the middle bolt in the fastener point cloud. The fastener coordinate system for the WJ-7 fastener is shown in Figure 11. The center of the middle bolt is the zero point, the X-axis is parallel to the rail track, and the Y-axis is perpendicular to the rail track. For a normal fastener, the relative position of the middle bolt and the fastener remain constant. However, in a real scenario, the coordinates of the fastener components do not always remain the same because the size of the isolation block varies, and sometimes the middle bolt is slightly skewed, which causes a change in the zero point. Therefore, key fastener components cannot be precisely positioned according to the design parameters.

Then, the point cloud of the key components has to be segmented from the point cloud of the fastener. Point cloud segmentation algorithm can be roughly classified into three types: region-growing based algorithms, feature-clustering based algorithms, and model-fitting based algorithms [27]. The key components of a fastener, such as the iron pad and the anchor bolt, are complex surfaces. Therefore, the model fitted-based algorithm is not suitable for this situation. Feature clustering-based segmentation algorithms require a large amount of computation and may have unwanted effects on real-time data processing. The region-growing-based algorithm is ideal for the segmentation of key component point clouds from fastener point clouds.

A modified region-growing algorithm is proposed to segment key component point clouds from the fastener coordinate system. Detailed steps regarding the development of the proposed algorithm are described in Algorithm 1. In this algorithm, “*SD*” denotes the initial seed point. The seed point is located according to the original design of the fastening system and the location of target components in fastener’s coordinate system. The height threshold, δ, is set to 0.2 mm. The variable $P_{n}$ represents the set output point, and $B_{p}$ represents the set boundary point. The variable $B_{i}$ represents a point in $B_{p}$. Eight points neighboring $B_{i}$ are added to $B_{p}$ and $P_{n}$ if the height deviation of $B_{i}$ is smaller than δ. The variable $B_{i}$ is erased from $B_{p}$ after the eight neighboring points are analyzed. Eventually, $B_{p}$ becomes empty, and the region-growing process terminates.

**Algorithm 1.** Modified region-growing algorithmInput: Seed point (*SD*)Output: Point set ($P_{n}$)Locate *SD*Check the eight neighboring points of the seed point, if their height compared to *SD* is smaller than that of the set threshold, δ, add them to $P_{n}$ and $B_{p}$Select a point, $B_{i}$, in $B_{p}$ and define its eight neighboring points as $T_{i}$.Check all of the points in $T_{i}$; if any point does not belong to $P_{n}$, and its height difference between $B_{i}$ is smaller than δ, add it to $B_{p}$ and $P_{n}$.Erase $B_{i}$ from $B_{p}$.Loop process 3 to 5 until $B_{p}=⊘$Output $P_{n}$

**Figure 12:**
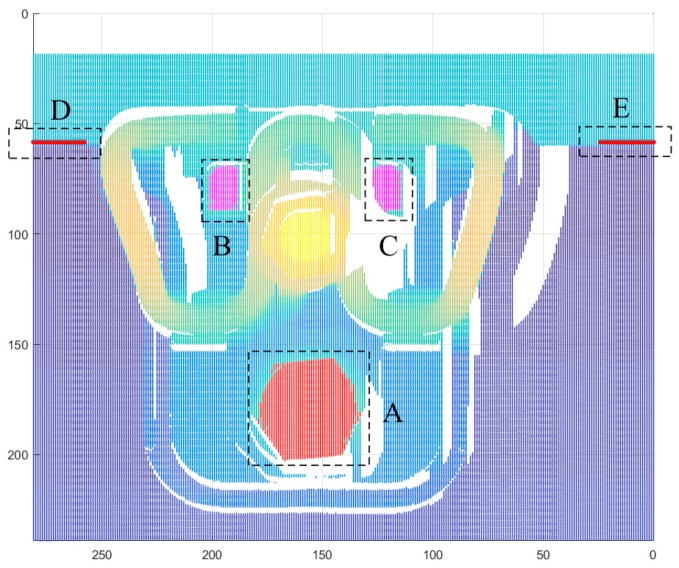
Extracted point clouds of the key components for a WJ-7 fastener.

Figure 12 shows the extracted point clouds of the key components for a WJ-7 fastener. The red point in dotted box A is the upper surface of the anchor bolt. The magenta points in dotted boxes B and C represent the upper surface of the iron plate limit pillar. The point clouds are extracted with the modified region-growing algorithm. The red points in dotted boxes D and E represent the edges at the bottom of the rail track. In the beginning and end frames of the fastener point cloud, the height of the points in a frame changes significantly at the bottom of the rail track, allowing the edge points to be found easily. Using these edge points and the standard model of a rail track, the rail track can be precisely located. For the WJ-8 or Vossloh-300 fastening systems, the key components should be selected according to the structure of the fastening system and geometric parameters of the target.

### 3.4. Geometric Parameter Measurement of the Fastener Components

Most components of the fastening system are fixed in size, but a few special components, such as the insolation block and height adjustment pads, have varying sizes. Based on the position of the key components and the structure of the fastening system, the geometric parameter of these special components can be measured indirectly. In this part, the geometric parameter measurement approach for WJ-7 fastener components is introduced. The approach for the WJ-8 and Vossloh-300 fasteners is based on the same principle.

**Figure 13:**
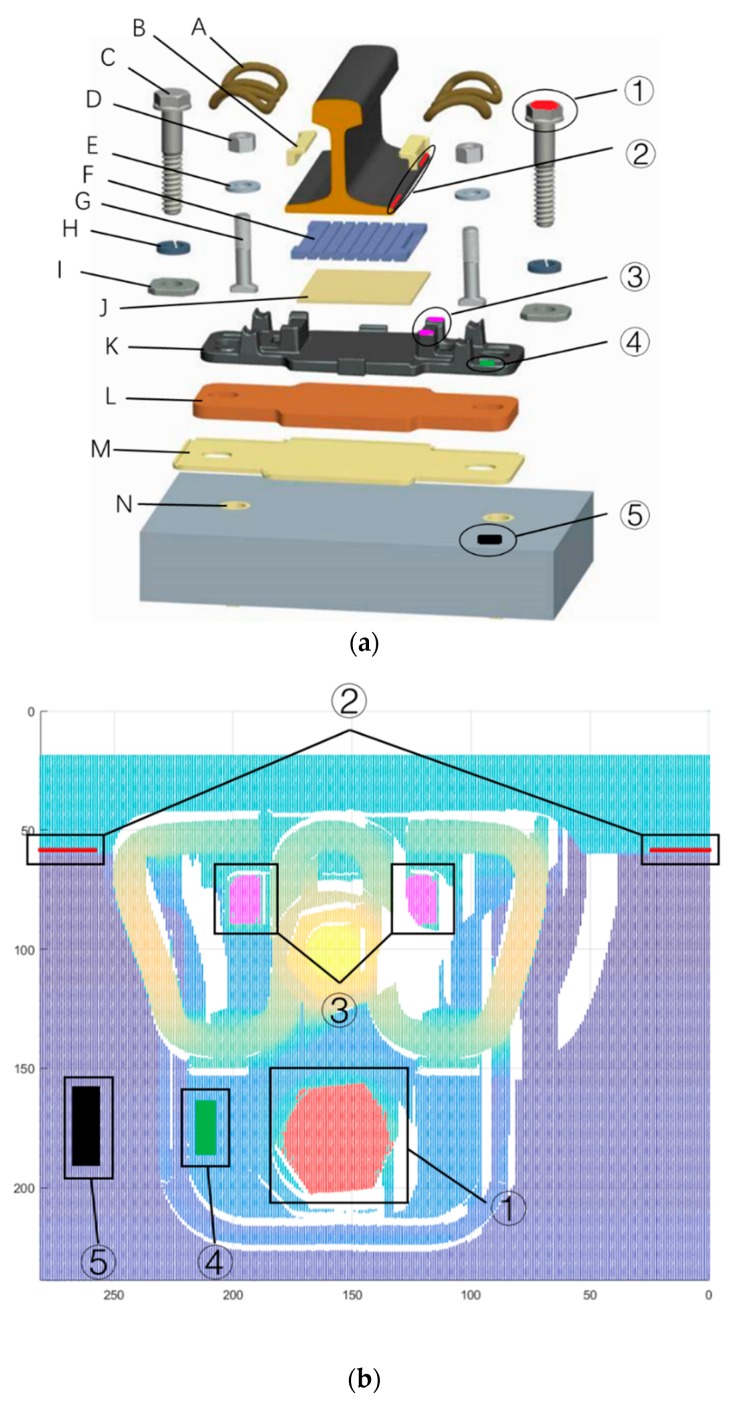
Geometric parameter measurements of the WJ-7 height adjustment pads: (**a**) detailed structure diagram of WJ-7 fastening system. (**b**) corresponding area of interest (AOI) in the point cloud. A: metal clip; B: insolation block; C: anchor bolt; D: nut; E: flat washer; F: rubber pad; G: T-bolt; H: spring washer; I: flat pad; J: height adjustment pad under the rail; K: iron plate; L: height adjustment pad under the iron plate; M: insolation and buffer pad; N: embedded bushing. ① Upper surface of anchor bolt; ② Rail bottom edge points; ③ Upper surface of the iron plate limit pillar; ④ Iron plate detection area; ⑤ Rail pad detection area.

The geometric parameter measurements of the WJ-7 height adjustment pads are shown in Figure 13. Figure 12 shows a detailed structure diagram of the WJ-7 fastening system, where all of the components are labeled, and the area of interest (AOI) to measure the pads are marked. The corresponding AOI in the point cloud is also marked in Figure 13b. 

There are two types of height adjustment pads in a WJ-7 fastening system, the height adjustment pad under the rail and the height adjustment pad under the iron plate. The height adjustment pad under the rail is placed under the rubber pad and above the iron plate. This pad has four specifications: 1 mm, 2 mm, 5 mm and 8 mm. The other height adjustment pad is placed under the iron plate. This pad has two specifications: 5 mm and 10 mm. Only the height adjustment pad under the rail is needed if the rail track should be lift up for less than 10 mm, otherwise, the height adjustment pad under the iron plate should be inserted as well. Up to two height adjustment pads under the rail can be used, but their total thickness should be less than 10 mm. With these height adjustment pads, rail tracks can be lifted up for a maximum of 30 mm. 

The thickness of the height adjustment pads under the rail can be calculated through Equation (4). In this equation, $T_{R}$ represents the thickness of the height adjustment pad under the rail; $h_{2}$ represents the height at the bottom edge of the rail track, which has been marked as ② in Figure 13; $h_{3}$ represents the upper surface height of the iron plate limit pillar, which is marked as ③ in Figure 13; $T_{Iron}$ represents the height difference between the upper surface of the iron plate limit pillar and the middle-upper surface of the iron plate (for a WJ-7 fastener, $T_{Iron}$ is 25.3 mm); $T_{Rail}$ represents the thickness of the rail track bottom (for a standard 60 kg/m rail track, $T_{Rail}$ is 12 mm); and $T_{Rubber}$ represents the thickness of the rubber pad ($T_{Rubber}$ is 11 mm).

(6) $$T_{R}=h_{2}-h_{3}+T_{Iron}-T_{Rail}-T_{Rubber}$$

The thickness of the height adjustment pad under the iron plate can also be calculated by Equation (5) with a similar principle. In the equation, $T_{P}$ represents the thickness of height adjustment pad under the iron plate; $h_{3}$ represents the upper surface height of the iron plate limit pillar, which is marked as ③ in Figure 13; $h_{5}$ represents the height of rail pad, which is marked as ⑤ in Figure 13; $T_{Pillar}$ represents the height difference between the upper surface of the iron plate limit pillar and the bottom of the iron plate ($T_{Pillar}$ is 35.1 mm); and $T_{Buffer}$ represents the thickness between the insolation and the buffer pads ($T_{Buffer}$ is 6 mm).

(7) $$T_{P}=h_{3}-h_{5}+T_{Pillar}-T_{Buffer}$$

The entire fastening system is fixed onto the rail pad by two hexagonal anchor bolts. The anchor bolts may loosen due to vibrations caused by the running train. In addition, rain water may seep into the embedded bushings and freeze in winter. The rain water will swell after forming into ice and push the anchor bolts out. This will aggravate the looseness of the anchor bolts. If the anchor bolts loosen, the fastener loses its anchoring affect and threatens the safety of the train. Therefore, it is important to detect whether or not the anchor bolt is loose. Currently, the looseness of the anchor bolt is inspected manually, but it can also be detected by analyzing the point cloud. If the anchor bolt is loose, the height difference between the upper surface of the anchor bolt and the edge of the iron plate increases. Therefore, the looseness of the anchor bolt can be worked out by Equation (6). In this equation, $T_{A}$ represents the looseness of the anchor bolt; $h_{1}$ represents the upper surface height of the anchor bolt, which is marked as ① in Figure 13; $h_{4}$ represents the height of the iron plate edge, which is marked as ④ in Figure 13 (this area is selected according to the location of iron plate limit pillar); and $T_{Checksum}$ indicates the height difference between $h_{1}$ and $h_{4}$ if the anchor bolt is fully tightened (for a fully tightened anchor bolt, $T_{Checksum}$ is 41.2 mm).

(8) $$T_{A}=h_{1}-h_{4}-T_{Checksum}$$

Occasionally, due to various reasons, horizontal rail track irregularity may occur. To maintain the standard gauge (1435 mm), rail tracks must move horizontally. Although the anchor bolts of the WJ-7 fastener are fixed onto the rail pad, the through holes in the iron plate are elliptical rather than circular, which enables the iron plate and rail track to move horizontally. The range of horizontal movement for the WJ-7 fastener is 6 mm. In some cases, the track adjustment plan fails if the fastener is horizontally and maximally displaced and has to move even more according to the plan. Therefore, the horizontal margin of the fastener is a key parameter. This can be worked out by calculating the distance between the rail bottom edge and the upper surface at the center of the anchor bolt.

In Section 3.1, the 3D posture of structured light sensors is calibrated. Therefore, the geometric parameter can be corrected through Equations (9) and (10), where α, β, γ denotes yaw, pitch, and roll pitch of the structured light sensors, *H* stand for thickness parameters and *R* represent horizonal parameters, $H_{c}$ and $R_{c}$ is *H* and *R* after correctness.

(9) $$H_{C}=H\cos\alpha \cos\gamma$$

(10) $$R_{C}=R\cos\beta$$

## 4. Experimental Results

In this section, the postures and relative positions of the structured light sensors are calibrated by the special made calibration blocks first. Then, field tests were conducted on a high-speed railway, and the geometric parameter of the fasteners was also measured manually to verify the results of the IRC.

### 4.1. Calibration Result of the Structured Light Sensors

Calibration is a critical process for measurement systems. The calibration block is placed under the rail track, its two ridges are placed parallel and symmetrical with the tail track, and the block are laid horizontally. The rail tracks that IRC runs on are smooth and without defects, and the top surface of the two rail tracks are checked by levels. The initial setup ensures that the IRC and the calibration block are all horizontal. One calibration block can calibrate two consecutive fasteners at the same time. Because there are four fasteners in a sleeper train, two identical calibration blocks are used. 

**Figure 14:**
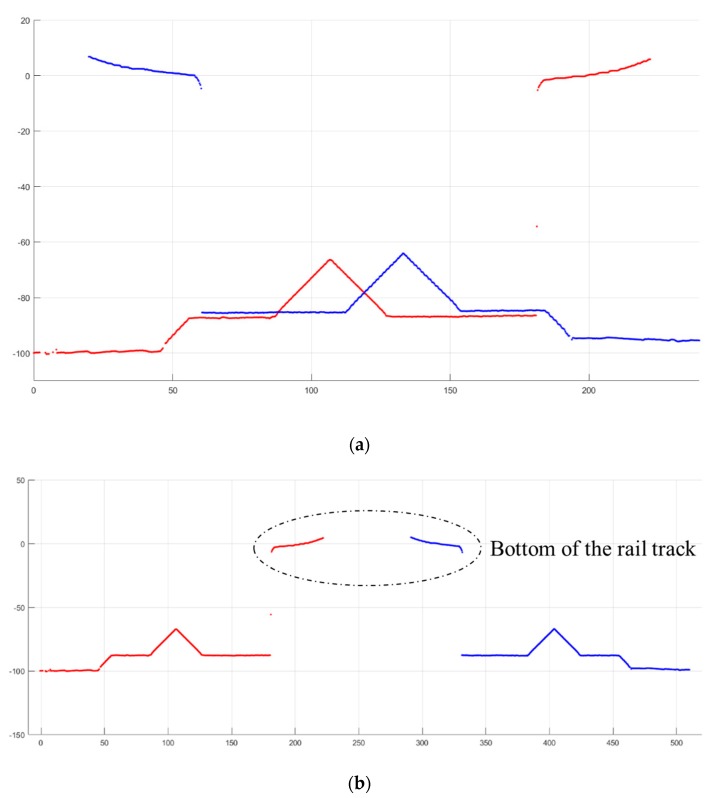
Calibration of the structured light sensors: (**a**) Original output of two consecutive sensors; (**b**) Side view of frames after calibration.

The calibration of the structured light sensors is shown in Figure 14. In this figure, the red point cloud is from the left sensor, and blue point cloud is from the right sensor. Points in upper half are bottom of the rail track and the rest points are ground and part of the calibration block. Figure 14a shows the original output point cloud for two consecutive structured light sensors. The point clouds of the two structured light sensors are placed into the same coordinate system. Figure 14b shows side view of the point cloud after calibration. The point cloud from the left sensor is maintained, and the point cloud from the right sensor is moved to match the design drawing of calibration block. The calibration results of the four structured light sensors are shown in Table 1. The sensor ID of the IRC is shown in Figure 4a. The horizontal and vertical positions represent the relative position between sensor 1 and 2, sensor 3 and 4. The relative position follows a right-handed coordinate system.

### 4.2. Result Verification of the Geometric Parameter Measurement

A field test was conducted on a high-speed railway near Wuhan, China. The fasteners in the test area were all WJ-7 fasteners. The test time was from 0:00 a.m. to 5:00 a.m., when high-speed trains were not running. The IRC was pushed along the rail track manually in a 500 m long test area. There were about 3400 fasteners in this area and the running speed of IRC is about 1 m/s, same as normal walking speed. The IRC was pushed forward and backward in the same area to test its repeatability in geometric parameter measurement. The whole calculation process including point cloud acquisition, fastener extraction, and geometric parameter calculation were all conducted in the single board computer (Intel ATOM D2550@1.86 GHz) of the system in real-time. In the same area, 100 fasteners were randomly selected (manually measurement of these geometric parameters was very time-consuming), and height adjustment pad under the rail, height adjustment pad under the iron plate, as well as the anchor bolt looseness, were measured manually for comparison with the IRC. The corresponding geometric parameters of fasteners measured by IRC were picked out.

**Figure 15:**
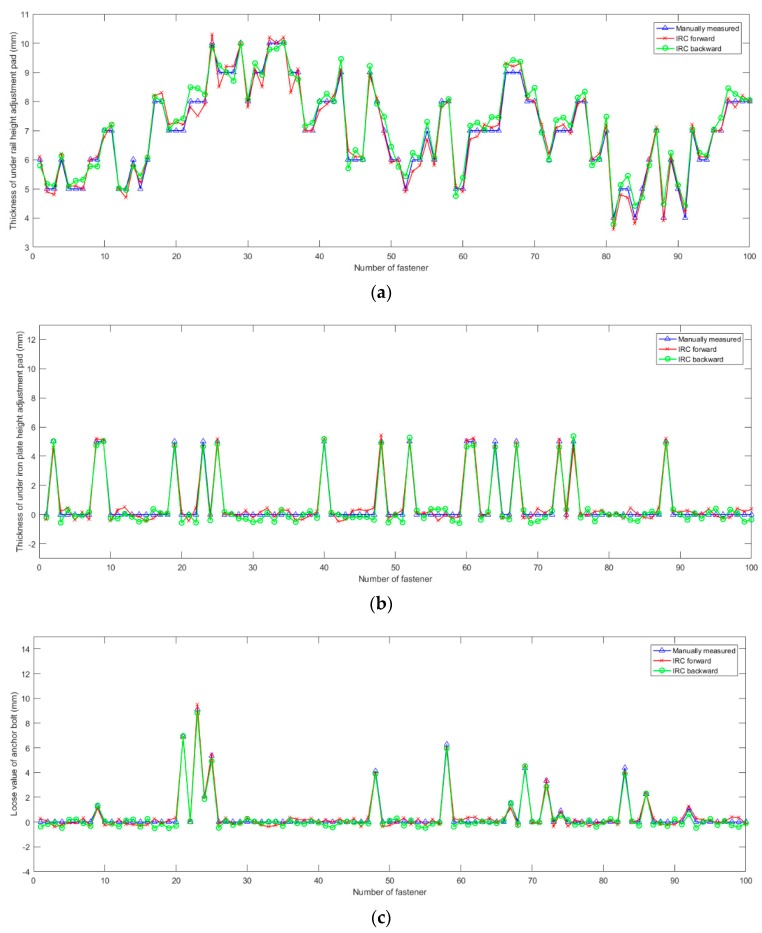
Geometric parameter results by manual measurements, IRC forward, and IRC backward. (**a**) Thickness of under rail height adjustment pad; (**b**) Thickness of under iron plate height adjustment pad; (**c**) Loose value of anchor bolt.

The geometric parameter results from manual measurements, IRC forward, and IRC backward are shown in Figure 15. In manual measurement, the thickness of the pad was obtained by identifying its specification. Therefore, the manually measured thicknesses were all integers. Since these 100 fasteners are randomly selected from the test area, the geometric parameters are distributed randomly. Figure 15a shows the result of thicknesses of height adjustment pad under the rail. Figure 15b shows the resulting thicknesses of height adjustment pad under the iron plate. The thickness of height adjustment pad under the iron plate had two specifications, 5 mm and 10 mm. If there was no height adjustment pad under the iron plate in the fastening system, the manual measurement result was 0. Since pads with a 10 mm thickness were not found among the selected fasteners, the manually measured thicknesses were either 0 or 5 mm. Figure 15c shows the resulting loosening values of the anchor bolts. If the anchor bolt of the fastener was not loose, the manually measured value was set to 0. 

Errors in geometric parameters between the manual measurements and the IRC are shown in Table 2. IRC forward and backward measurement results are compared with manual measurement respectively. The maximum error in the geometric parameter measurement was 0.7 mm, and the maximum root mean square error (RMSE) was 0.3 mm. The results show that the IRC was precise when measuring the geometric parameter of the fastener. In addition, the IRC can show the results in real-time when being pushed along a high-speed railway. It took 2 operators 30 min to measure the geometric parameters of 100 WJ-7 fasteners, however, the IRC measured these fasteners in 15 s. 

## 5. Conclusions

In this paper, a real-time geometric parameter measurement system for high-speed railway fasteners based on structured light sensors is presented. Commercial structured light sensors Keyence LJ-V7000 are used, and the layout of the system is explained in detail. Dense and precise 3-D point clouds of the high-speed railway fasteners are obtained from the system. Using a special made calibration block, the 3D postures and relative positions of the consecutive structured light sensors are calibrated. A fastener extraction method is proposed to extract the fasteners point cloud. An eight-neighbor region-growing algorithm is presented to locate key components. Then, the geometric parameters of WJ-7 fasteners are calculated according to the location of the key components and the structure of the fastener. An experiment is conducted on a high-speed railway near Wuhan, China to verify the accuracy and repeatability of the system. The result shows that the maximum RMSE between the manual measurement and the system is 0.3 mm, which demonstrates adequate accuracy (smaller than accuracy requirement, 0.5 mm) and good repeatability in the geometric parameter measurement of WJ-7 fasteners. This system can replace manual measurements and greatly improve the efficiency of geometric parameter measurements for fasteners. 

## Figures and Tables

**Table 1:** Calibration result of the four structured light sensors.

Sensor ID	Yaw (α)	Pitch (β)	Roll (γ)	Horizontal Position	Vertical Position
1	0.08°	0.12°	0.32°	0	0
2	0.09°	0.08°	0.45°	271.21 mm	−1.71 mm
3	0.11°	0.06°	0.57°	0	0
4	0.06°	0.12°	0.40°	275.26 mm	−2.58 mm

**Table 2:** Errors in the geometric parameter between manual measurements and the IRC.

Geometric Parameter	Maximum Error (mm): Forward/Backward	Minimum Error (mm): Forward/Backward	Mean Error (mm): Forward/Backward	RMSE (mm): Forward/Backward
Thickness of height adjustment pad under the rail	0.6/0.5	−0.7/−0.6	0.1/0.1	0.2/0.2
Thickness of height adjustment pad under the iron plate	0.6/0.6	−0.6/−0.7	0/0.1	0.3/0.3
Loose value of anchor bolt	0.4/0.4	−0.5/−0.6	0.1/0.1	0.1/0.1
